# Azaporphyrins Embedded on Carbon-Based Nanomaterials for Potential Use in Electrochemical Sensing—A Review

**DOI:** 10.3390/nano11112861

**Published:** 2021-10-27

**Authors:** Tomasz Koczorowski, Magdalena Cerbin-Koczorowska, Tomasz Rębiś

**Affiliations:** 1 Chair and Department of Chemical Technology of Drugs, Poznan University of Medical Sciences, Grunwaldzka 6, 60-780 Poznan, Poland; 2 Department of Medical Education, Poznan University of Medical Sciences, 7 Rokietnicka Str., 60-806 Poznan, Poland; mcerbin@ump.edu.pl; 3 Institute of Chemistry and Technical Electrochemistry, Poznan University of Technology, Berdychowo 4, 60-965 Poznan, Poland; tomasz.rebis@put.poznan.pl

**Keywords:** phthalocyanines, porphyrazines, graphene, carbon nanotubes, sensing

## Abstract

Phthalocyanines and porphyrazines as macrocyclic aza-analogues of well-known porphyrins were deposited on diverse carbon-based nanomaterials and investigated as sensing devices. The extended π-conjugated electron system of these macrocycles influences their ability to create stable hybrid systems with graphene or carbon nanotubes commonly based on π–π stacking interactions. During a 15-year period, the electrodes modified by deposition of these systems have been applied for the determination of diverse analytes, such as food pollutants, heavy metals, catecholamines, thiols, glucose, peroxides, some active pharmaceutical ingredients, and poisonous gases. These procedures have also taken place, on occasion, in the presence of various polymers, ionic liquids, and other moieties. In the review, studies are presented that were performed for sensing purposes, involving azaporphyrins embedded on graphene, graphene oxide or carbon nanotubes (both single and multi-walled ones). Moreover, possible methods of electrode fabrication, limits of detection of each analyte, as well as examples of macrocyclic compounds applied as sensing materials, are critically discussed.

## 1. Introduction

Electrochemical biosensors have gained wide acceptance in diagnostics due to their advantages as simple, real-time, rapid, and economic systems. The biosensors possessing high selectivity, sensitivity, and low detection limit (even picomolar) are in the scope of interest of the chemical and pharmaceutical industry. The electrode materials play an essential role in fabricating high-performance electrochemical sensing platforms for detecting target compounds. Several materials for modifying electrodes, such as carbon-based nanomaterials, polymers, metal nanoparticles, and silica nanostructures or their hybrids, have been widely used [1,2,3]. Carbon-based nanomaterials (CBNs) present unique electrochemical properties, including high effective surface area, excellent electrical conductivity, electrocatalytic activity, and adsorption capability, making them potential candidates for electrochemical sensing [4]. Graphene hybrid composites based on their honeycomb lattice and carbon nanotubes with embedded inorganic nanostructures, conducting polymers, and other organic materials have been widely used to fabricate electrochemical sensors of biomolecules, where they exhibited enhanced electrochemical activity.

Due to the fact that both graphene and CNTs are electron-conductive materials, their film on an electrode can increase the effective area available to the analyte. For this reason, the sensitivity of the response towards various analytes can be elevated. Furthermore, CNTs reveal an electrocatalytic effect on the oxidation of many analytically essential substances, including hydrogen peroxide, NADH, dopamine, ascorbic acid, uric acid, and norepinephrine [5].

**Figure 1:**
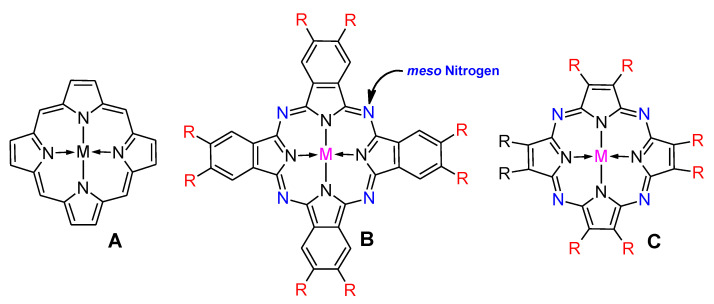
The chemical structure of porphyrin (**A**), phthalocyanine (**B**) and porphyrazine (**C**) rings. “R” stands for peripheral substituents and “M” indicates metal cation inside core.

The modification of electrode surfaces enhancing their sensing properties can also be obtained by different porphyrinoids [6,7]. This large group of macrocyclic complexes consists mainly of porphyrins (Ps), phthalocyanines (Pcs), and porphyrazines (Pzs) (Figure 1). Pcs and Pzs can be classified as azaporphyrins due to the presence of *meso* nitrogen atoms in place of methine bridges in the porphyrin macrocyclic ring [8], impacting their unique physicochemical and electrochemical properties [9]. All porphyrinoids possess an extended π-electron system that allows them to undergo fast redox processes. Among the electrode modifiers reported in the literature, porphyrinoids were considered as powerful electrocatalysts for the electrochemical determinations of many biologically important compounds, such as hydrogen peroxide, ascorbic acid, and L-glutathione [6,10]. The electrocatalytic properties were dependent on the central metal ion (e.g., Co, Fe, Mn, Ni), as well as on substituents attached to macrocyclic rings on their peripheries [11]. Peripheral substituents also affect basic physicochemical properties of porphyrinoids, like their solubility.

Many efforts have been made for the last 15 years to use azaporphyrins, particularly phthalocyanines, in electrochemical sensing [12]. In most studies, the sensing material consisting of macrocyclic compounds was immobilized on the surface of carbon-based nanomaterials, like carbon nanotubes and graphene. The fabricated hybrid nanomaterials were further used for working electrode modification. There are several methods of conjugation between azaporphyrins and carbon-based nanomaterials [7]. The most abundant rely on non-covalent interaction involving π–π stacking between azaporphyrin and CBN. This can be achieved by drying the solution of the macrocycle on the nanomaterial (“drop-dry” technique) or by immersing electrodes modified with CBNs in the Pcs solution (“dip-dry” technique). Other methods consist of layer by layer electrostatic assembly [13], immobilization in conducting polymer matrix [14], in situ cyclotetramerization of macrocycle on the surface of carbon-based nanomaterial [15] or grinding in mineral oil [16]. The covalent attachment can be driven by i.e., amide formation between carbocyclic acid-functionalized CBNs and azaporphyrin with peripheral amine or sulfonic substituents [17].

Herein, we present a comprehensive review of the electrochemical determination of various analytes with the use of working electrodes modified with both graphene or carbon nanotubes and embedded metallic azaporphyrins. For a clearer analysis, the article is divided into sections presenting the detection of distinct groups of analytes.

## 2. Food Pollutants and Components Sensing

Nowadays, agriculture and food production use a wide variety of chemical compounds that increase the speed and efficiency of production. Starting with fertilizers used in plant cultivation, through drugs administered to farm animals, ending with food additives, we are exposed to the consumption of many toxic substances. High concentrations of some chemicals, used in food manufacturing, can be hazardous to human health and cause serious toxic disorders. For this reason, the detection and concentration determination of food pollutants is a challenging task for public health protection.

Several electrochemical sensing methods were employed to determine some food contaminants or nutrients in real samples. The sulfite ion-selective electrode based on unsubstituted cobalt(II) phthalocyanine non-covalently embedded on multi-walled carbon nanotubes (MWCNTs) was proposed by Abd-Rabboh et al. Sulfite is an commonly used preservative agent in beverages. The modified screen-printed carbon electrode manifested higher selectivity and sensitivity towards sulfite ions in comparison to standard iodometric method [18]. Other widespread preservative agents are citrates and their salts. For their electrochemical determination, the surface of glassy carbon electrode (GCE) was modified with monoamiono-substituted zinc(II) phthalocyanine covalently attached to graphene oxide [19]. The fabricated sensor revealed in potentiometric studies a 500 nM limit of detection of citrate and a linear range of 0.8 µM–10 mM.

On the other hand, determining some naturally occurring compounds in food and beverages is highly desirable due to their health benefits. For example, polyphenols like catechins are strong antioxidizing agents present in green tea. The determination of their concentration in green tea extracts is an important issue due to increasing level of dietary supplements consumption with catechins [20]. Given this, an electrochemical biosensor based on tyrosinase accompanied by unsubstituted iron(II) phthalocyanine adsorbed on the surface of SWCNTs was employed for measuring the polyphenols in vegetable samples and green tea extracts [21]. The obtained tyrosinase-screen-printed single-walled carbon nanotubes (SWCNTs) electrode biosensor presented a low detection limit and short analysis time compared to a classical Folin-Ciocalteau analytical method. The samples of green tea and another popular beverage, cola, were examined to determine caffeine and its derivative theophylline. In the studies performed by Koçak et al., the symmetrically substituted manganese(III) phthalocyanine with tetra 5-chloroquinolin-8-yloxy groups at the peripheral positions/MWCNTs system embedded on the surface of GCE was used to determine the aforementioned purines by differential pulse voltammetry [22]. The extended π electron system of macrocycle by addition of the quinolone peripheral substituents in this case definitely increased the intermolecular interaction between azaporphyrin and nanotubes enhancing the electrocatalytic ability of the sensing material obtained. In other studies, multi-walled carbon nanotubes deposited with unsubstituted cobalt(II) phthalocyanine on the surface of carbon paste electrode was used in determination of the essential components of the diet-polyunsaturated fatty acids (PUFAs). The modified electrode demonstrated a specific applicability towards various PUFAs, especially linoleic acid, and was applied in a flow injection analysis system for the determination of PUFAs in a complex matrix and commercially available safflower oil [23].

The large group of food pollutants are pesticides. These are natural or synthetic organic compounds. Pesticides can be divided on several groups like herbicides, insecticides, fungicides, repellents etc. High levels of some pesticides can be extremely harmful to the central nervous system and cause serious damage. For this reason, various analytical techniques, mostly based of chromatography methods were used in determination of pesticides in food samples [24]. Several examples of phthalocyanines/CNTs modified electrodes were used in electrochemical sensing of diverse pesticides like fenitrothion, methyl parathion, deltamethrin, chlorpyrifos, spinosad, metolcarb, fenobucarb, carbaryl, physostigmine, carbamate pesticide formetanate hydrochloride, herbicide glyphosate and insecticide imidacloprid [24,25,26,27,28,29,30,31,32,33]. The surface of working electrode (GCE or screen-printed carbon electrode) was covered by MWCNTs in most studies with one exception of using reduced graphene oxide or graphene nanoribbons instead of nanotubes [27,31,32]. Both substituted and unsubstituted metal phthalocyanines (M = Ru, Cu, Co, Zn, Ni, Mn) were employed as a sensing material attached to the surface of carbon-based nanomaterial by covalent [24] and non-covalent bonding. The carbon-based material was also supported by silica nanoparticles [25], Nafion [30] and a copper complex [33] to enhance the electrocatalytic performance of obtained recognition element. The combination of the carbon nanostructures and phthalocyanines with extended π-systems enhance the accumulation of aromatic pesticides by hydrophobic interaction. As a result, more sensitive determination of pesticides is possible. Moreover, both transition metal and noble metal-based phthalocyanines are highly efficient electron mediators. Such a unique property enables the decrease of high overpotentials of many organic compounds including pesticides. Acceleration of electron transfer provides oxidation of pesticides at beneficial potential with limited interference. In all cases, the prepared modified electrodes revealed excellent performance in real food samples i.e., in determination of fenitrothion, methyl parathion and physostigmine in orange juice [24,25,33], metolcarb and fenobucarb in spiked vegetable samples [26,27], formetanate hydrochloride in mango and grape samples [30], imidacloprid in honey samples [32], and carbofuran in carrots [31].

## 3. Gas Sensing

Gas sensing devices are widespread in modern industry, wherever there is a need to detect and warn about threats to human health and life. Besides, a high concentration of some gases can also cause damage to industrial equipment and even lead to industrial disasters. Gas sensors are commonly used to determine the presence of polluted gas in factories and laboratories. Recently, various toxic gases (i.e., NH_3_, SO_2_, and H_2_S) have been released due to rapid developments and unforeseen accidents in various industries. Therefore, a technology to detect gaseous air pollutants and toxic gases has become very important. The development of efficient and fast electrochemical gas sensors will meet these expectations due to their low cost, high sensitivity and selectivity. The gas sensing devices based on semiconducting materials rely on electrochemical resistance in a presence of an analyte adsorbed on sensing material.

One of most toxic gases widespread in the food and chemical industry is ammonia (NH_3_). In humans, its perception by smell can be achieved at the level of approximately 50 ppm. However, when its concentration exceeds 25 ppm, ammonia is very harmful and can lead to serious damage of the respiratory tract and cause eye and skin irritation. Great effort has been made to develop new sensing materials, regarded as a key problem in the fabrication of high-performance ammonia sensing devices. In the last 10 years over a dozen examples of utilizing azaporphyrins adsorbed on carbon-based materials as a semiconducting layers were presented and evaluated for the determination of ammonia. Metal phthalocyanines provide specific gas-sensing active sites for selective NH_3_ adsorption. Changes in conductivity upon exposition to NH_3_ make metal phthalocyanines an attractive class of ammonia-sensing materials. Chemiresistor sensing responses can be correlated with the Lewis basicity and hydrogen bonding strength between gas molecules and metal phthalocyanine. The aforementioned interactions strongly depended on the central metal atom in Pcs (M: Co, Ni, Cu, Zn). Moreover, electron affinity of metal centre plays important roles in interactions between gases and metal phthalocyanines [34]. What is important is that the unique planar 18 π-electron-conjugated structure of azaporphyrins allows them to be closely coupled with the nanostructured carbon by π–π interactions. On the other hand, carbon provides fast and efficient charge transfer. Hence, the gas-sensing performance of metal phthalocyanine/carbon hybrids can be significantly improved.

**Figure 2:**
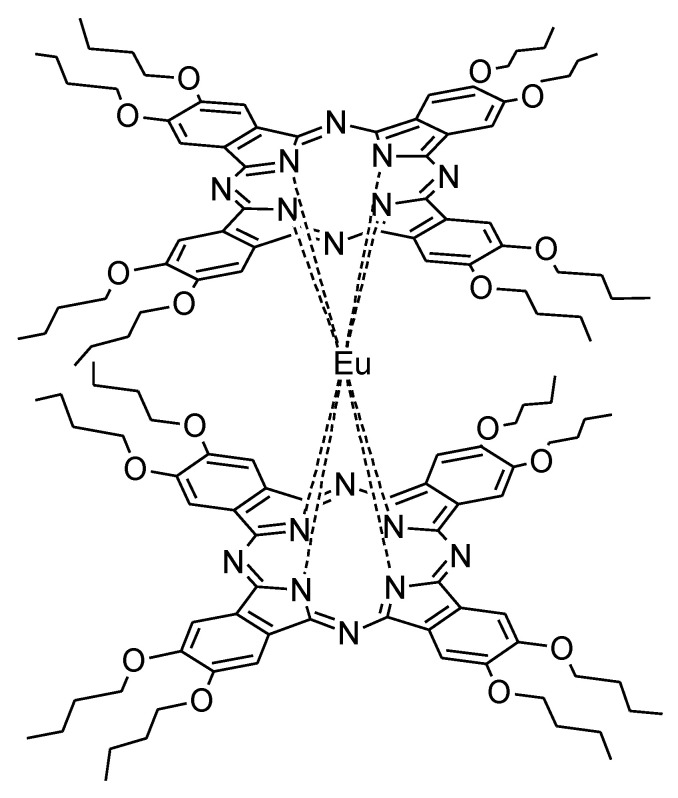
The chemical structure of the double-decker europium(II) phthalocyanine peripherally substituted with eight buthoxy groups [35].

An interesting sensing material for determination of both NH_3_ and nitrogen dioxide was obtained by Abdullah et al. The double-decker europium(II) phthalocyanine peripherally substituted with eight buthoxy groups (Figure 2) was non-covalently attached to acidified multi-walled carbon nanotubes. The fabricated semiconducting sensor achieved a limit of detection (LOD) of 0.5 ppm for ammonia and 0.3 ppm for nitrogen dioxide [35].

**Figure 3:**
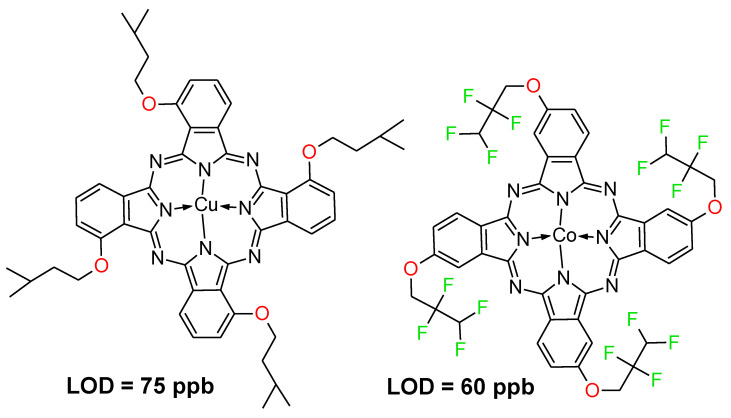
The chemical structures of two phthalocyanines with the lowest limit of detection (LOD) in ammonia sensing [39,40].

Multi-walled and single-walled carbon nanotubes were used as a semiconducting support for preparation several examples of ammonia sensing devices based on unsubstituted zinc(II) and aluminium(III) phthalocyanines [36] and cobalt(II) phthalocyanine [37]. Various substituted phthalocyanines were employed in fabrication of ammonia sensors. Bonegardt et al. assessed fluoroalkyl (ZnPc-CF) and alkyl (ZnPc-CH) substituted sulfanyl zinc(II) phthalocyanines embedded on SWCNTs and concluded that the first one exhibited twice less sensor response time, whereas the second was more stable at higher humidity [38]. Comparative studies of a series of copper, nickel, lead tetra-α-isopentyloxyphthalocyanines non-covalently adsorbed on the surface of MWCNTs were performed by Kang et al. The low limit of detection (75 ppb) accompanied by a recovery time of 180 s was achieved in the case of tetra-α-isopentyloxy substituted copper(II) phthalocyanine [39]. However, the lowest LOD (60 ppb) was obtained for cobalt(II) phthalocyanine with peripheral 2,2,3,3-tetrafluoropropoxy substituents embedded on non-functionalized MWCNTs (Figure 3) [40]. Such a low LOD might be a result of introducing epoxy substituents in the periphery of macrocycles in comparison to unsubstituted or alkyl substituted phthalocyanines due to the stronger impact of oxygen electron lone pairs on the ring π-electron system involved in ammonia sensing. Also, the presence of fluorine substituents enhanced the limit of detection obtained.

Following this phenomenon, in other studies, Kaya et al. showed better efficiency in the detection of ammonia in case of copper(II) phthalocyanine asymmetrically substituted with polyoxyethylene groups and bearing one pyrene group in comparison to a cobalt(II) analogue [41]. Despite the impact of a central metal cation on the sensing efficiency of phthalocyanines, the size of the aromatic ring and the structure of the peripheral substituents were also important. When the phthalocyanine ring was displaced by naphthalocyanine (Nc) and deposited on SWCNTs, it displayed better response due to extended π-electron system resulting in deposition of more macrocyclic molecules in comparison to Pcs [42]. In the case of peripheral substitution, two types of copper(II) phthalocyanines with α-iso-pentyloxy and α-2,2,4-trimethyl-3-pentyloxy substituents were deposited on SWCNTs and used as ammonia sensors by Wang et al. [43]. The better response was achieved by the sensing material based on the first copper(II) phthalocyanine with a less developed periphery. The same research team evaluated the impact of type of carbon-based nanomaterial on the determination of ammonia. The lead(II) 2,9,16,23-tetra-iso-pentyloxyphthalocyanine was non-covalently attached to the surface of acidified single- and multi-walled carbon nanotubes. This time, the SWCNTs hybrid system revealed slightly better sensing properties [44]. However, to enhance this properties by MWCNTs, their surface can be doped by metal oxides alloy [45] or can be integrated with polyaniline (PANI) [46].

The determination of ammonia with the use of sensing material consisting of metal phthalocyanine was also assessed when the macrocyclic compound was embedded on graphene and its reduced form, and on nitrogen-doped graphene [47,48,49,50,51]. In the last case, the copper(II) tetrasulfophthalocyanine supported on three-dimensional nitrogen-doped graphene based frameworks was integrated with poly(3,4-ethylenedioxythiophene)-poly(styrenesulfonate) (PEDOT-PSS) to obtain sensing film. Such combined nanocomposite possessed the enhanced sensing properties than hybrid material composed of phthalocyanine and graphene [47]. 

**Figure 4:**
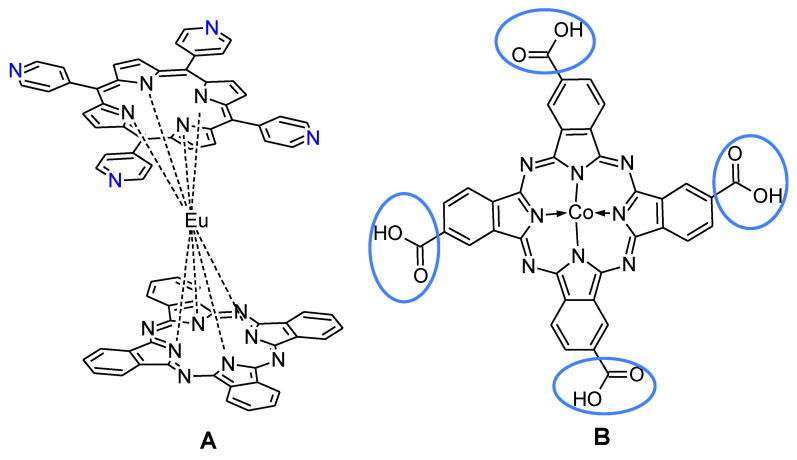
The chemical structure of the sandwich type double decker phthalocyanine/porphyrin europium(II) complex (**A**) [55] and tetracarboxylic cobalt(II) phthalocyanine (**B**) [55].

Another important and very dangerous gas is nitrogen oxide. It is produced from vehicle exhaust and as a product of some combustion processes. NO_2_ requires a special monitoring as a result of its harm to health and the environment. It can cause respiratory tract disorders when its concentration exceed 80 ppb. In the fabrication of nitrogen oxide gas sensing devices based on azaporphyrins, graphene derivatives were the most used semiconducting materials. However, Brunet et al. used a mixture of carbon nanocones and nanodiscs/powdered indigo/MWCNTs hybrid material and unsubstituted Cu(II)Pc to obtain high efficient resistive sensor of NO_2_ [52]. In another exception example, unsubstituted copper(II) phthalocyanine was deposited by π–π stacking on hydroxylated SWCNTs and high selectivity in the thin film resistors was observed [53]. Zhu et al. used a sandwich type double decker phthalocyanine/porphyrin europium(II) complex (Figure 4A) in situ self-assembled on the surface of reduced graphene oxide to obtain hybrid aerogel. The limit of detection of nitrogen dioxide of a prepared sensor was theoretically estimated on 80 ppb [54]. The lower LOD (50 ppb) was achieved when tetracarboxylic cobalt(II) phthalocyanine was deposited on the surface of graphene quantum dots (Figure 4B) [55]. Once again, the introduction to electron-reach peripheral substituents might have an influence on obtaining lower LOD by increasing the π–π stacking interaction of the macrocycle ring and nanomaterial. On the other hand such a bulky structure sandwich-type complex can hamper the access of the analyte to the recognition element.

When describing the electrochemical nitrogen oxide sensors, it is worth mentioning that azaporphyrins/CNT sensing materials were employed not only for detection of gaseous NO_2_ or some nitrites in aqueous samples but also for detection of nitrates produced in vivo by living cells. Various nitrates are commonly used as food additives, dyeing agents, and corrosion inhibitors. From the biomedical point of view they play a key physiological role in signaling, blood flow regulation, hypoxic nitric oxide homeostasis, and cancer. For this reason, it is important to monitor in situ the level of nitrogen oxide released from cells and tissues. For instance, unsubstituted iron(II) phthalocyanine was embedded by non-covalent interaction on the surface of nitrogen-doped graphene. The biocompatibility of such a sensing material was obtained by following layer-by-layer assembly of poly-L-lysine (PLL) and Nafion [56]. Some other interesting examples of real-time determination of nitrates produced by living cells were performed by the use of iron porphyrins as a building blocks of needle microsensors [57], field-effect transistors [58] and covalent organic frameworks [59]. In the case of azaporphyrins, a first sensor for the amperometric determination of nitrates was based on unsubstituted cobalt(II) and iron(III) phthalocyanines deposited on MWCNTs and used for surface modification of glassy carbon or screen-printed carbon electrodes [60,61]. In the latter example, the poly-3,4-ethylenedioxythiophene (PEDOT) covered the surface of MWCNTs, to enhance the electrostatic interaction between the negatively charged nitrite and oxidized PEDOT film. The cobalt(II) phthalocyanine with 3-trifluoromethylphenoxy substituents was embedded on non-functionalized MWCNTs by π–π stacking interaction. The obtained hybrid material was used to modify the surface of a glassy carbon electrode, which showed fast electron transfer rate and excellent electrocatalytic activity for the oxidation of nitrites [62]. Not only phthalocyanines but also porphyrazines were used in the electrochemical determination of nitrites. The symmetrical substituted sulfanyl nickel(II) and manganese(III) porphyrazines accompanied by SWCNTs were used for the electrocatalytic oxidation of nitrites and the results showed that the electrocatalytic current for nitrite oxidation is promoted well when Ni(II)Pz was used [63].

**Figure 5:**
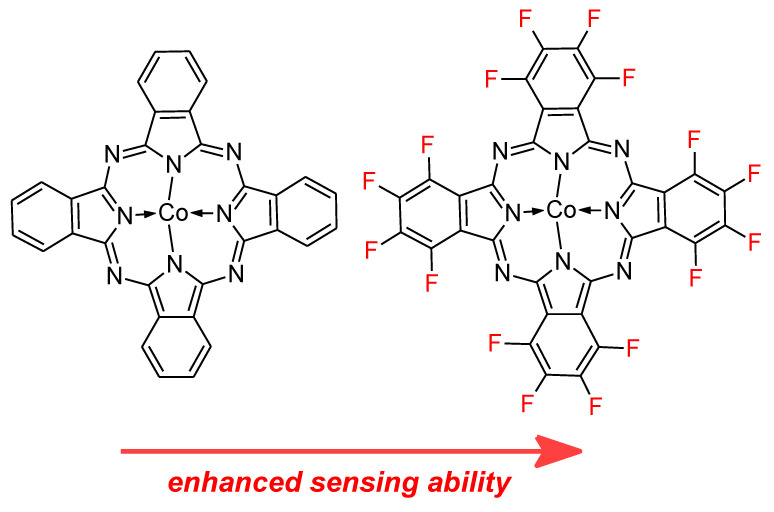
The chemical structures of unsubstituted and hexadecafluorinated cobalt(II) phthalocyanines.

Chlorine is a gaseous pollutant that is of interest for sensing devices based on phthalocyanines and carbon-based nanomaterials. Chlorine is widely used in pharmaceutical and agrochemical industry, for water treatment, textile production, and it is often present in household cleaning products. As a result of a serious health risk which can be caused by Cl_2_, there is a need to develop sensing devices with a ppb range of detection. There are some examples of Pcs/CBN able to manage this issue. For instance, hexadecafluorinated and unsubstituted cobalt(II) phthalocyanines were deposited on reduced graphene oxide and exhibit LOD of 1.41 and 1.97 ppb, respectively [64,65], indicating better sensing activity when strong electron withdrawing substituents were introduced into the macrocyclic ring (Figure 5). Furthermore, in 2017 and 2018 Sharma et al. performed several studies using hexadecafluorinated zinc(II), copper(II), cobalt(II) phthalocyanines non-covalently embedded on the surface of carboxylic acid functionalized single-walled and multi-walled carbon nanotubes in terms of their ability to detect ppb levels of chlorine. This particular case revealed a strong influence of central metal cation inside the macrocyclic core on the sensing ability of recognition element. The lowest LOD = 0.04 ppb was achieved in case of cobalt(II) phthalocyanine and SWCNTs-COOH in comparison to 0.27 ppb for copper(II)Pc and 0.06 for zinc(II)Pc [66,67,68,69].

**Figure 6:**
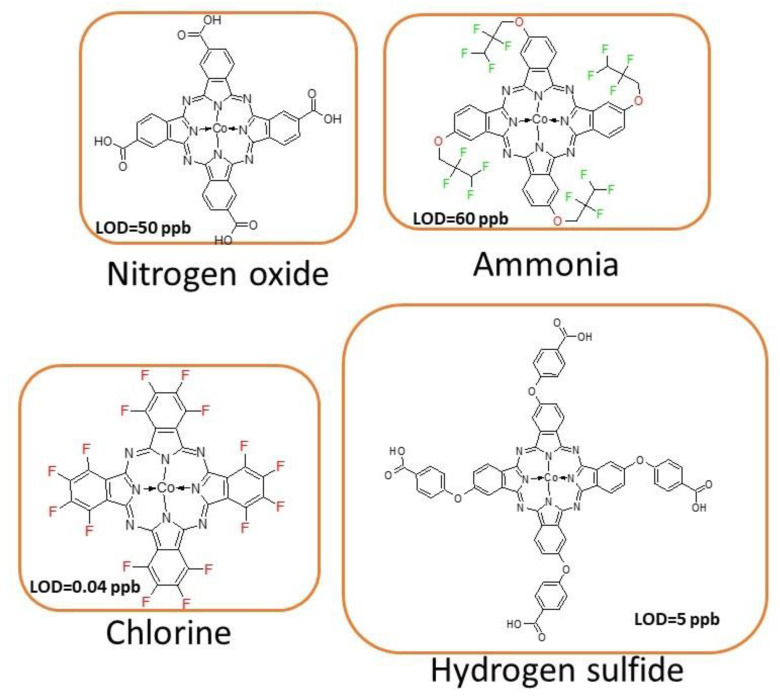
The chemical structures of phthalocyanines possessing lowest LOD in determination of selected gases.

Among common air pollutants, hydrogen sulfide (H_2_S) is a widespread toxic and flammable gas, produced in biodegradation processes in the petroleum industry and as a by-product of bacterial decomposition of human and animal feces. What is more, H_2_S can be present in humans’ exhaled breath and plays a crucial role in the diagnosis of various diseases like diabetes, asthma, lung cancer. In such cases, the ppb level of limit of detection should be reached by potential sensing devices for their promising application in medicine. This goal has been achieved. Wang et al. adsorbed 4-trifluoromethylphenoxy substituted Co(II)Pc on reduced graphene oxide and estimated LOD of 11.6 ppb [70]. Better results (LOD = 5 ppb) were obtained when cobalt(II) phthalocyanine peripherally substituted by carboxyphenyloxy substituents was covalently attached to acidified MWCNTs with the use of hydroquinone, *p*-aminophenol, and *p*-phenylenediamine as linking molecules [71]. Figure 6 presents the chemical structures of most active phthalocyanines used in determination of the aforementioned gases.

For safety in the laboratory, another issue is fast detection of volatile organic compounds (VOCs) like toluene, benzene or xylene. All of them were a matter of interest in studies performed by Ndiaye et al. The tetra-*tert*-butylo substituted copper(II) phthalocyanine in combination with phenyl and alkyl *meso*-substituted porphyrins were non-covalently attached to the surface of single-walled carbon nanotubes and assessed towards VOCs like BTX (benzene, toluene, xylene) in four studies. The nature of peripheral moieties of sensor and analyte desorption mechanisms and kinetics were investigated. A significant improvement of the sensor responses due to the high surface area of the nanomaterial was observed [72,73,74].

An interesting analyte (dimethyl methylphosphonate, DMMP) was examined by Wang et al. in 2011 and by Jiang et al. in 2021 [75,76]. DMMP is a nerve agent stimulant—a simulant of sarin. Sarin is considered as a chemical weapon (chemical warfare agents, CWAs) due to the fact that it is an extremely toxic synthetic organophosphorus compound. It was used in a terrorist attack on the Tokyo subway in 1995, in which 13 people were killed and thousands poisoned. Therefore, timely detection of CAWs such as sarin is a very important issue in public safety and military fields. Unsymmetrical substituted cobalt(II) phthalocyanine was non-covalently attached to graphene quantum dots. The fabricated DMMP sensor revealed LOD of 500 ppb [76]. The same macrocyclic compound was non-covalently attached to SWCNTs and the obtained sensor also revealed excellent sensitivity towards DMMP [75].

## 4. Detection of Heavy Metals in Water Samples

Water pollution is one of the major environmental problems in both developing and modern countries. In many cases some toxic substances are detected in water and food samples and in this way they can be very harmful to human beings. Especially heavy metals are risky and hazardous to human health with the possible development of allergies, tumors and serious central nervous system diseases. Thus, monitoring their levels in surface and drinking water should be performed with care. Among many toxic metals, cadmium and lead are the most widespread. Nowadays spectrometric and chromatographic techniques are used for determination of Cd^2+^ and Pb^2+^ ions in water samples. Recently, some new electrochemical methods have been developed including glassy carbon electrodes with adsorbed nickel oxide. However, in 2019, Arnold-Tatu et al. and van Staden et al. published their work about utilization of unsubstituted iron(II) and nickel(II) phthalocyanines deposited on reduced graphene oxide and graphene paste, respectively [77,78]. The obtained modified electrodes were assessed in determination of lead and cadmium ions in water samples by using the coordination abilities of porphyrinoids. The results showed that the sensing ability of a modified electrode was twice higher in Pb^2+^ determination in comparison to an unmodified electrode [77].

## 5. Environmental Pollutant Sensing

An important environmental pollutant is phenol and its derivatives—bisphenol A and 2-aminophenol. They are intermediates or by-products of industrial production of plastics, chemical dyes and some pharmaceutics. Due to their easy access to the human body through the digestive system and skin penetration, phenol and its derivatives possess toxic effects including DNA damage and damage to reproductive capability. Generally, the level of phenols is determined by time consuming mass spectrometry and gas and liquid chromatography. Therefore, efforts have been made to develop faster electroanalytical techniques. De Oliveira et al. fabricated an efficient biosensor based on tyrosinase, gold nanoparticles, MWCNTs and cobalt(II) phthalocyanine for determination of phenol in wastewater [79]. Tetrasulfonic acid substituted zinc(II) phthalocyanine was adsorbed on oxidized exfoliated graphite powder by an electrostatic self-assembly method and deposited on GCE. Similar to carboxyl substituents, the sulfonic moieties increased the ability of macrocycle adsorption on the surface of the nanomaterial and elevated electronic conductivity. The modified electrode revealed very good sensitivity towards bisphenol A [80]. Moreover, recently Jilani et al. modified GC electrode with sorbamide substituted cobalt(II) phthalocyanine deposited on MWCNTs and used it for determination of 2-aminophenol obtaining low LOD of 0.016 µM and linear range of 0.1–38 µM with differential pulse voltammetry (DPV) measurements [81].

Among phenol environmental pollutants, catechol is also one of most abundant. It is widely produced by the chemical, cosmetic and pharmaceutical industry. Due to its high toxicity and low degradability, the levels of catechol should be monitored in water and soil samples. Despite chromatographic techniques, an electrochemical detection of catechol and its derivatives was also considered and studied. Silva et al. fabricated glassy carbon electrode with multi-walled carbon nanotubes modified by unsubstituted manganese(II) phthalocyanine and used it in amperometric sensing of catechol and hydroquinone [82]. Another type of carbon-based nanomaterial—reduced graphene oxide (rGO) was used by Wu et al. [83]. The methylated cobalt(II) tetra-β-(N,N-diethylaminoethoxy)phthalocyanine was deposited on rGO to form thin films by layer-by-layer assembly. The obtained sensor revealed much better LOD of catechol in comparison to MWCNTs-based samples due to an increased recognition area. The electrochemical determination of catechol, hydroquinone, phenol and *p*-nitrofenol by the use of unsubstituted Co(II)Pc embedded on fulvic acid reduced GO and deposited on GCE was performed by Zhang et al. [84]. The best electrocatalytic performance of a modified electrode was achieved in case of catechol sensing. 4-nitrophenol was also determined by the use of unsubstituted iron(II) phthalocyanine embedded on graphene oxide, fabricated from graphite through graphite oxide and deposited on GCE. The modified electrode was used for electrochemical detection of the analyte in human urine samples [85]. Cesarino et al. fabricated another electrochemical sensor of various phenol derivatives as a byproducts formed after the electrolysis of benzene. The glassy carbon electrode revealing the micromolar limit of detection of each analyte was modified by deposition of MWCNTs with unsubstituted Co(II) phthalocyanine on their surface [86].

Another dangerous environmental pollutant is hydrazine. This highly toxic reducing agent, widely used in the chemical and pharmaceutical industry, can cause serious damage to human beings, affecting skin, kidneys, liver and DNA. Hydrazine was also subjected to electrochemical sensing studies, involving either substituted cobalt(II) phthalocyanine or porphyrazines embedded on reduced graphene oxide or MWCNTs, respectively [87,88]. The first sensor revealed nanomolar limit of detection due to extended π-electron system of phthalocyanine in comparison to porphyrazine [87]. An interesting modification of glassy carbon electrode was performed by Mani et al. where the surface of the electrode was covered by dispersion of both graphene and carbon nanotubes accompanied by unsubstituted iron(II) phthalocyanine [89]. In this case, a nanomolar LOD (93 nM) and linear range of 0.5–83.5 µM were obtained.

## 6. Biomarkers Sensing

### 6.1. Peroxides

Hydrogen peroxide is a natural substance that is present in every body. Its presence is very beneficial to organism—it is necessary to support metabolic processes of proteins, carbohydrates, fats, as well as minerals and vitamins. Furthermore, it participates in the enzymatic and hormonal systems, and also supports the transfer of glucose from the blood plasma to cells (insulin independent transport). Hydrogen peroxide also has a detoxifying function. It oxidizes some harmful toxins, and causes damage of bacteria and viruses. With the help of the catalase, it turns into atomic oxygen and water. However, it is also known for its cytotoxic effects in living organisms. Despite hydrogen peroxide, some organic peroxides i.e., *tert*-butyl hydroperoxide (TBHP) also play an important role in biological issues like ageing and mutagenic processes. Standard analytical methods for determination of various peroxides are based on fluorimetry, chemiluminescence, and gas chromatography. Due to their simplicity, electrochemical methods were extensively developed. As a result of high overpotential in the reduction of peroxides, chemically and enzymatically modified electrodes should be used. However, their stability is difficult to maintain. For this reason, an immobilization of sensing material with the use of various polymers like polyvinylferrocenium was performed [90].

Several azaporphyrins deposited on carbon-based nanomaterials were used in electrochemical determination studies of peroxides. High sensitivity of azaporphyrins towards hydrogen peroxide based on the electrocatalytic activity of transition metal centre (e.g., Co or Mn). The electrocatalytic mechanism of metallic porphyrazines can be considered as the oxidation of H_2_O_2_ to O_2_ with parallel reducing M(III)Pz to M(II)Pz. To preserve the catalyst’s activity, M(II)Pz is subsequently electrochemically oxidized back to M(III)Pz. Electrocatalytic activity for the reduction of H_2_O_2_ is also possible when metal azaporphyrins are used as electrocatalyst. In such a case H_2_O_2_ is reduced to H_2_O by oxidizing M(I)Pz to M(II)Pz, and M(II)Pz can be reduced back to M(I)Pz at the electrode surface [91].

There are many examples in the literature revealing the synergistic effect of various carbon nanomaterials combined with azaporphirins toward peroxides electrocatalysis. For example, cobalt(II) and manganese(III) tetraaminophthalocyanines were covalently attached to phenylamine functionalized SWCNTs, deposited on the surface of screen-printed gold electrode and used as hydrogen peroxide sensors with good results [92]. However, in comparison to other studies described below, the covalent attachment seems to be less effective than non-covalent interaction based on π–π interaction due to a lower amount of macrocyclic molecules in the recognition element. Other functionalized SWCNTs were used by Pillay et al. in the electrochemical detection of H_2_O_2_. The layer-by-layer technique was used in fabrication of thin films consisted of poly(*m*-aminobenzenesulfonic acid) (PABS) functionalized SWCNTs—iron(II) phthalocyanine. These films were deposited on a gold electrode [93]. The modified electrode reveals more amplified reduction of H_2_O_2_ when the number of layers increased. Zhang et al. used the same electrostatic layer-by-layer (LBL) deposition technique to modify glassy carbon electrode with a water-soluble cationic phthalocyanine and acidified MWCNTs [94]. Hydrogen peroxide was also the subject of determination studies with the use of dendrimeric sulfanyl magnesium(II) porphyrazine non-covalently attached to MWCNTs and deposited on GCE [95].

**Figure 7:**
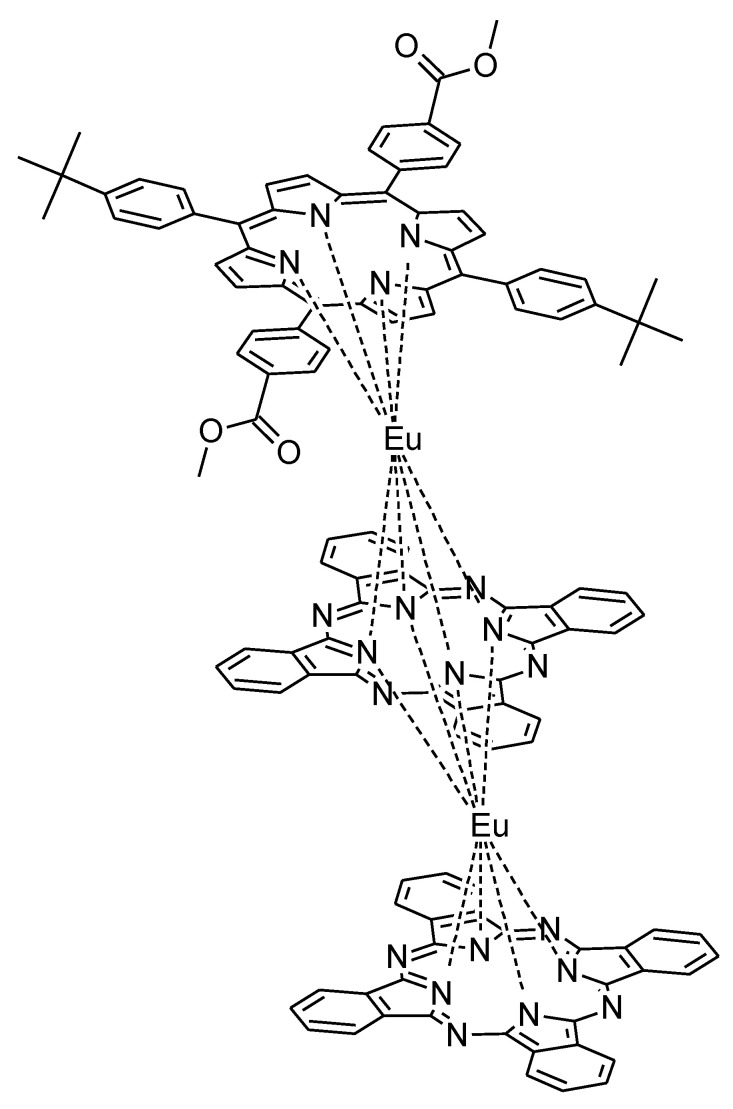
The chemical structure of the triple-decker phthalocyanine-porphyrin europium(III) complex [97].

Despite the use of carbon nanotubes, some graphene sheets and graphene oxide accompanied by azaporphyrins have been used in H_2_O_2_ sensing. For instance, Gorduk et al. fabricated tetra-(4-oxy-3-methoxyphenyl)acrylic acid copper(II) phthalocyanine—graphene modified pencil graphite electrode for this purpose [96]. The introduction of acrylic moieties is in this case an another example of π-electron system expansion useful in the electrochemical reduction of hydrogen peroxide. In addition, an interesting sensing material was used in studies performed by Yu et al. where a triple-decker phthalocyanine-porphyrin europium(III) complex (Figure 7) was non-covalently attached to graphene oxide and multi-layer hybrid films were obtained [97].

*Tert*-butyl hydrogen peroxide was detected with good sensitivity and long-term stability on modified GC electrode by Jin et al. with the use of unsubstituted iron(II) phthalocyanine non-covalently attached to reduced graphene oxide, doped by Fe_3_O_4_ nanoparticles [98]. The same analyte was the subject of determination studies performed by Cui et al., in which unsubstituted cobalt(II) phthalocyanine was embedded on PF_6_-functionalized graphene [99].

### 6.2. Thiols

L-Cysteine is one of the endogenic amino acids, which combined with L-glycine and L-glutamine forms one of the most powerful antioxidants—glutathione. The level of glutathione is considered as a key factor responsible for life expectancy. L-cysteine can support the body’s natural fight against reactive oxygen species (ROS). The excess of ROS causes oxidative stress—a dangerous condition, where healthy cells from all over the body are destroyed. The high level of L-cysteine in various tissues helps prevent serious diseases such as ulcerative colitis, cancer, atherosclerosis, and male infertility.

The chemical modification of electrodes used in the electrochemical determination of L-cysteine and other biothiols is necessary to hamper the overpotential obtained on the bare electrode. The oxidation of the SH group of cysteine to form the disulfide L-cystine is known to be catalyzed by metal ions such as Fe(III) or Co(II). According to the previous study the catalytic mechanism involves the adsorption of thiol at the catalytic centre and the creation of the intermediate adduct [Fe(II)-RS]_ads_ [100]. The electrocatalytic properties of azaporphyrins used in thiols sensing were enhanced rather with the use of reduced graphene oxide than with carbon nanotubes. In two similar studies peripheral substituted sulfanyl iron(II) and cobalt(II) porphyrazines were non-covalently attached to rGO and deposited on GCE [101,102]. The modified electrodes revealed high sensitivity and their sensing ability was not influenced by common plasma interferences like glucose, uric and ascorbic acids. Reduced graphene oxide was also used by Mani et al. in L-cysteine determination studies with the use of tetraamino substituted cobalt(II) phthalocyanine [103]. Despite L-cysteine, other biothiols like glutathione were detected by unsubstituted copper(II) and manganese(II) phthalocyanines deposited on rGO or graphene oxide [104]. Biothiols were also employed as a sensing analytes in the studies with the use of cobalt(II) phthalocyanine deposited on nitrogen-doped graphene [105]. Covalent attachment of tetranitro substituted cobalt(II) phthalocyanine on graphene oxide was obtained by Hosseini et al. and revealed excellent analytical features in the determination of L-cysteine on modified GCE [106]. On the other hand, non-covalent attachment of unsubstituted Co(II)Pc on multi-walled carbon nanotubes functionalized with carboxyl groups was used in the preparation of modified GC electrode in determination of glutathione and its reduced form as an oxidative stress biomarkers [107]. Luz et al. fabricated in 2010 a modified basal plane pyrolytic graphite (BPPG) electrode for determination of glutathione in erythrocytes [108]. The surface of BPPG disc in Teflon^®^ was covered by a mixture of tetrasulfo-substituted cobalt(II) phthalocyanine and iron(III) tetra-(N-methyl-4-pyridyl)-porphyrin embedded on MWCNTs. The analogous azaporphyrin, tetrasulfo-substituted iron(II) phthalocyanine non-covalently attached to unmodified MWCNTs was used to modify a glassy carbon electrode for electrochemical determination of L-cysteine [109].

### 6.3. Catecholamines

Catecholamines form a group of similar hormones produced in the adrenal medulla. The essential catecholamines like dopamine, adrenaline (epinephrine) and noradrenaline (norepinephrine) are released into the blood in response to physical and emotional stress. Under normal conditions, catecholamines and their metabolites are present in the body in small, periodically variable amounts, and their concentrations increase noticeably only during and shortly after experiencing stress. Pheochromocytomas and other neuroendocrine tumors can produce large amounts of catecholamines, which increases the amount of these hormones and their metabolites in the blood and urine. Catecholamines secreted by phaeochromocytoma can cause persistent hypertension, headaches, arrhythmias, sweating, nausea, and restlessness.

**Figure 8:**
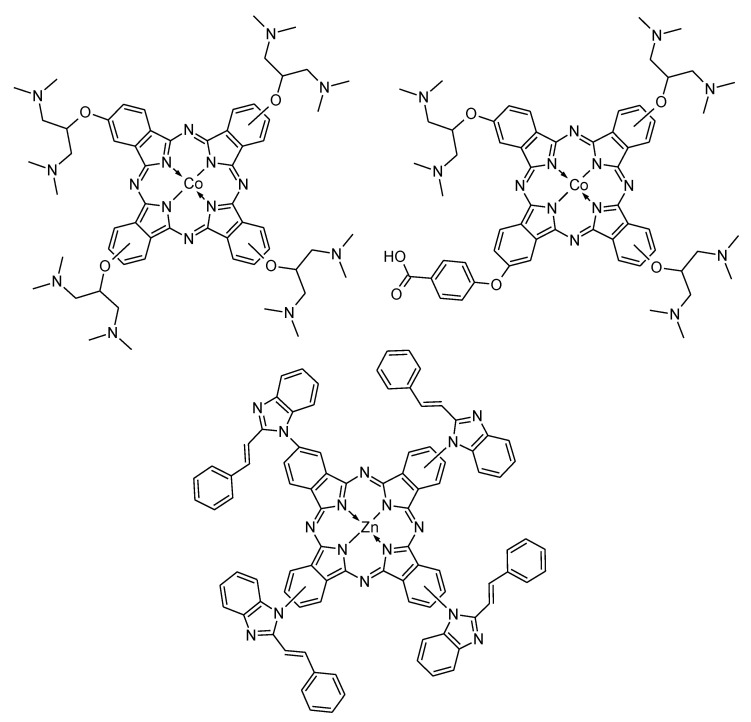
The chemical structures of symmetrical and unsymmetrical cobalt(II) and zinc(II) phthalocyanines investigated by Ndebele et al. and Pari et al. [119,120].

Dopamine was the most subjected analyte in the electrochemical determination of catecholamines with the use of CBN-modified electrodes. Several studies were performed using working electrodes, modified by symmetrical and unsymmetrical metal phthalocyanines, and embedded both on carbon nanotubes and graphene. The well-known two-proton, two-electron electrochemical oxidation of dopamine can be accelerated by the transition metal centre in azaporphyrins. The catalytic electrooxidation of dopamine is related to the reversible and fast redox transition of M(III)/M(II) couple [110]. Siqueira et al. and Xu et al. used tetrasulfonated nickel(III) phthalocyanine, non-covalently attached to PAMAM-modified MWCNTs or nitrogen-doped graphene, respectively. The obtained sensing materials were used in determination of dopamine in presence of some interferences like ascorbic and uric acid. The modified electrodes revealed the limit of detection lower than 100 nM [111,112]. Much lower LOD (0.87 nM) and the linear range of 20 nM to 220 nM were obtained when a similar azaporphyrin—tetrasulfonated cobalt(II) phthalocyanine—was deposited on graphene sheets and used in the fabrication of surface sensing material by Diab et al. [113]. Unsubstituted cobalt(II) phthalocyanine was the most frequently used sensing macrocycle embedded on multi-walled and single-walled nanotubes for modification of various types of electrodes (i.e., poly(dimethylsiloxane)—mineral oil-modified carbon paste electrodes) in dopamine determination [114,115,116,117]. In one case, the conducting properties of carbon material was enhanced by platinum nanoparticles, however the calculated LOD was higher in comparison to that mentioned above [117]. Patrascu et al. performed the determination studies of dopamine in the presence of serotonin in deproteinized serum samples with the use of electrodes modified by unsubstituted iron(II) phthalocyanine and MWCNTs. The obtained results revealed large separation (approx. 170 mV) between dopamine and serotonin peak potentials. The gap was enough for the simultaneous determination of both substances in the same media [118]. Graphene quantum dots and reduced graphene oxide were also used as electron amplifiers in determination studies of dopamine, performed by Ndebele et al. and Pari et al., respectively [119,120]. In these studies various symmetrical and unsymmetrical cobalt(II) and zinc(II) phthalocyanines were used (Figure 8). In the case of ZnPc, the very low limit of detection of 6 nM was obtained accompanied by linear range of 20 nM to 1 µM and sensitivity of 2.8784 µA µM^−1^ cm^−2^ [120].

Despite dopamine, another catecholamine, epinephrine, was subjected to two electrochemical studies using unsubstituted cobalt(II) phthalocyanine embedded on MWCNTs or edge-plane pyrolytic graphite-single walled carbon nanotubes [16,121]. The obtained LOD was 15.6 nM and 40 nM, respectively.

### 6.4. Other Biomarkers (NADH, Ascorbic Acid, Uric Acid and Urea, Nucleic Acids and Nucleotides)

There are numerous examples of electrochemical sensing of other biomarkers like ascorbic and uric acids, nucleic acids and nucleotides. The reduced β-nicotinamide adenine dinucleotide (NADH) is the key compound in energy production in living cells. As a coenzyme, more than 300 dehydrogenases-based enzymatic reactions involve NADH. The direct oxidation of NADH on conventional electrodes occurs in high overpotentials, which leads to passivation of the electrode surface. Therefore, its electrochemical determination was performed with the use of iron(II), magnesium(II) and nickel(II) sulfanyl porphyrazines embedded on reduced graphene oxide or SWCNTs [102,122]. Peripheral groups of porphyrazine can act as an electrocatalyst of NADH oxidation. In one of the works, 2,3,7,8,12,13,17,18-octakis{2-[2-(4-nitrophenoxy)ethoxy]ethylthio}porphyrazinato magnesium(II) were deposited on the surface of SWCNT and subsequently electro activated by cyclic voltammetry. According to the study, the nitro groups, being part of porphyrazine substituents, can be electrochemically reduced forming hydroxylamine moieties and next transformed to o-aminophenol/o-iminoquinone groups. The electrocatalytic effect for NADH oxidation was ascribed to the reaction of NADH with oxidized mediator (o-iminoquinone fragment) [122]. Reduced graphene oxide was also covered by tetrasulfonated nickel(II) phthalocyanine [123]. However, the obtained LOD was two times lower in the case of Pz (0.16 µM) than Pc (0.3 µM).

Ascorbic acid, well known as vitamin C, is a common anti-oxidant, which plays a key role in human metabolism as a free radical scavenger. Its high plasma levels are considered in prevention of free radical induced diseases like some cancers and Parkinson’s disease. In electrochemical determination studies involving azaporphyrins and carbon-based nanomaterials, three different types of metal phthalocyanines—copper(II) with peripheral sodium tetrasulfonic groups, and two cobalt(II)—and unsubstituted and tetra-neopentyloxy substituted Pcs were adsorbed on graphene or MWCNTs, respectively [15,124,125]. The lowest nanomolar limit of detection of ascorbic acid was achieved when graphene was used as a carbon-based material [124].

In the electrochemical determination of various analytes like L-cysteine, NADH or dopamine, there are some interferent species added during measurements to assess the selectivity of obtained modified electrode towards proper substrate. The common interfering compounds present in blood plasma are uric acid, ascorbic acid and glucose. However, there were studies where urea, uric and ascorbic acids were subjects of electrochemical determination but not as interfering compounds. Salvarajan et al. fabricated a modified GC electrode by deposition graphene oxide with unsubstituted zinc(II) phthalocyanine adsorbed on GO surface [126]. The electrode presented nanomolar limit of detection of urea. Uric acid was determined by the use of unsubstituted cobalt(II) phthalocyanine absorbed on MWCNTs and deposited on GCE with a LOD of 0.26 mM [127]. In the studies performed by Manjunatha et al., a glassy carbon electrode, modified through a self-assembled monolayer technique with tetraamino-substituted palladium(II) phthalocyanine adsorbed on MWCNTs, was used in the determination of three analytes: uric acid, dopamine and ascorbic acid in urine samples [128]. The obtained LODs were 1.50 µM, 0.60 µM and 1.00 µM, respectively.

Electrochemical determination was also employed for DNA sensing. The guanine oxidation response signal was analyzed. In the studies of Balan et al., a significant amplification of peak current was obtained for carbon nanotube paste electrodes modified with unsubstituted cobalt(II) phthalocyanine [129]. The obtained LOD for determination of single-stranded DNA by DPV was 98.6 nM. In other studies, the glassy carbon electrode was sequentially modified by MWCNTs-Co(II)Pc nanocomposite and PAMAM dendrimer (generation 4.0). The modified electrode was further employed as a DNA electrochemical biosensor of the nucleotide sequence related to genotype of the avian influenza virus [130]. In this case, the DNA strand was immobilized with the use of a dendrimer on the surface of electrode and the guanine oxidation peak current was measured in the absence or presence of a complementary target.

**Figure 9:**
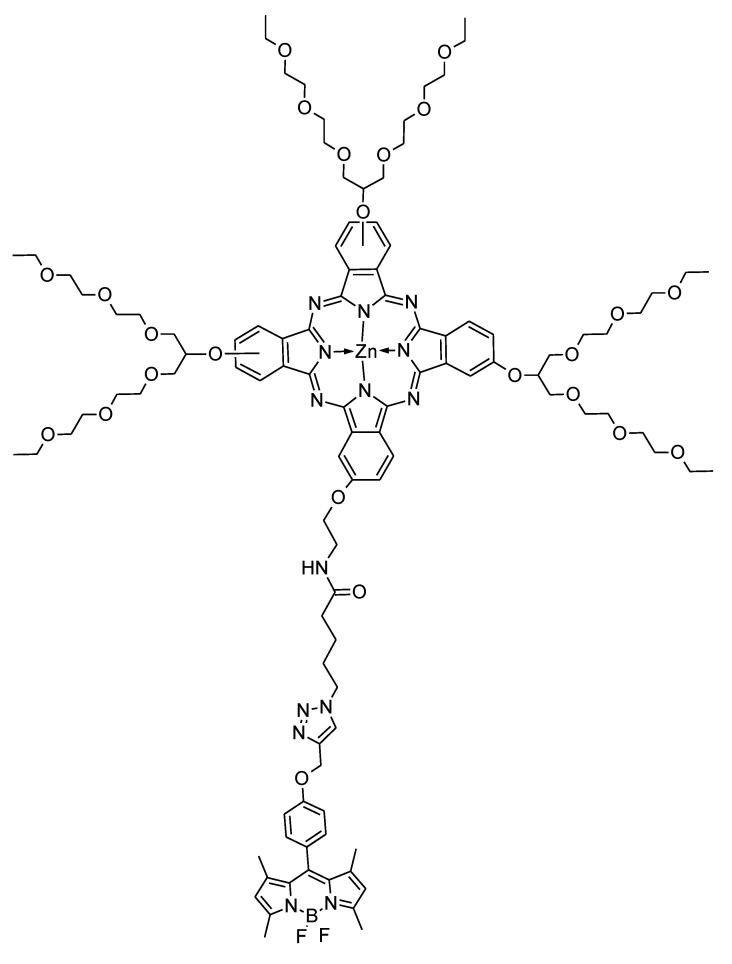
The chemical structure of the conjugate of substituted zinc(II) phthalocyanine and BODIPY dye [132].

Metal phthalocyanines were also used in combination of carbon-based materials in electrochemical determination of markers of prostate and colon cancers. The unsymmetrical substituted cobalt(II) phthalocyanine adsorbed on the surface of graphene quantum dots and covered by aptamer was embedded on a glassy carbon electrode for the electrochemical detection of prostate-specific antigen (PSA) [131]. Al-Ogaidi et al. used the conjugate of substituted zinc(II) phthalocyanine and BODIPY dye (Figure 9) for fabrication of an electrochemical sensor based on graphite paste. The determination of KRAS, a genetic marker of colon cancer, was performed in comparison to two electrodes modified with graphene oxide/TiO_2_ and azulenes [132].

A different type of carbon-based nanomaterial—graphene-like carbon nitride (g-C_3_N_4_)—was used by Dai et al. in the photoelectrochemical determination of choline. The sensing material was prepared through axial coordination of carbon nitride nanosheets to dendritic zinc(II) phthalocyanine [133].

## 7. Glucose Sensing

**Figure 10:**
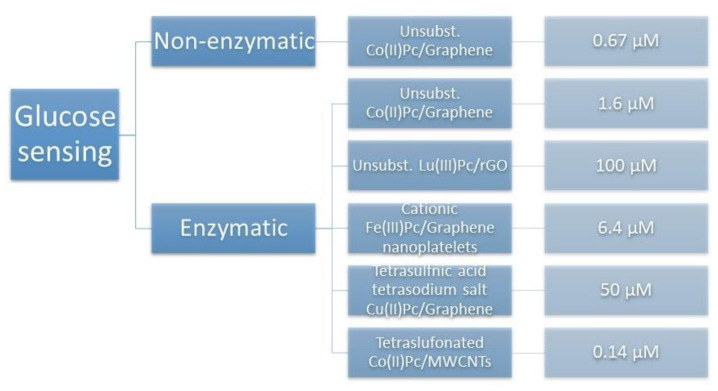
Examples of recognition elements based on azaporphyrins/carbon-based nanomaterials (CBNs) used in glucose determination with the obtained limits of detection.

Fast glucose detection in blood samples is a crucial issue in diagnosis and effective treatment of diabetes mellitus. Amperometric glucose sensing is based on the enzyme—glucose oxidase and its immobilization in matrix deposited on the surface of electrodes [134]. However, an non-enzymatic paper-based glucose sensor was developed by Chaiyo et al. where cobalt(II) phthalocyanine was embedded on graphene and deposited on a screen-printed carbon electrode [135]. Proposed analytical device reached wide linear concentration range of glucose (1.3 to 5.0 mM for a high concentration glucose assay) and LOD of 0.67 µM. Among the enzymatic sensors, to date a vast number of materials have been used for glucose oxidase immobilization, like: carbon-based nanomaterials, conducting polymers, cellulose acetate, polyacrylonitrile [136,137,138,139]. Several attempts were made with the use of azaporphyrins as a recognition supporting materials in the fabrication of new glucose electrochemical biosensors. Most of them were adsorbed on graphene. Unsubstituted cobalt(II) phthalocyanines non-covalently attached to graphene and graphene-polyaniline nanocomposites were used for immobilization of glucose oxidase for the amperometric detection of glucose in three different studies [91,140,141,142]. 1.6 µM was the lowers obtained limit of detection accompanied by very wide linear concentration range (10 µM to 14.8 mM) and high sensitivity of 5.09 µA mM^−1^ cm^−2^ [140]. In the case of graphene-polyaniline conducting material, a new type of compact disc microfluidic biosensor was fabricated and assessed towards glucose and hydrogen peroxide sensing [141]. Al-Sagur et al. performed two studies using lutetium phthalocyanine-rGO and water-soluble cationic iron(II)Pc-graphene nanoplatelets for immobilization of glucose oxidase [142,143]. In the second case, the lower LOD of 6.4 µM was obtained with linear range of 1 to 20 mM and sensitivity of 18.11 µA mM^−1^ cm^−2^. Another water-soluble azaporphyrin—tetrasulfnic acid tetrasodium salt copper(II) phthalocyanine—was adsorbed on graphene and immobilized with Nafion and glucose oxidase on the negatively charged surface of a glassy carbon electrode [144]. The obtained biosensor was used for electrochemical determination of glucose with a LOD of 50 µM (with a linear range up to 8 mM), based on oxygen consumption during glucose oxidation. In one study, multi-walled carbon nanotubes were used instead of graphene, accompanied by tetrasulfonated cobalt(II) phthalocyanine. The prepared enzyme-free glucose sensor exhibit very low limit of detection of 0.14 μM with the linear response from 10 µM to 6.34 mM and with the sensitivity of 122.5 µA mM^−1^ cm^−2^ [145]. A summary of particular phthalocyanines used in glucose sensing is presented in Figure 10.

## 8. Active Pharmaceutical Ingredients (APIs) Sensing

In terms of human health, improvement of quality of life is related to the increasing number of drugs taken by patients, including substances that may threaten their health and life. A vast amount of medication, taken mainly by older people, implies a risk of overdose and determines the need for therapeutic monitoring of their body concentration. Therapeutic drug monitoring (TDM) is the clinical practice of measuring the concentration of specific drugs at designated intervals to maintain their constant level in the blood for optimizing the individual dosage schedule. TDM is used mainly for monitoring drugs with narrow therapeutic ranges, drugs with marked pharmacokinetic variability, and medications for which target concentrations are difficult to monitor [146]. The increased number of APIs and their dosage forms makes it necessary to improve the therapy’s control in many drugs. Thus, specific, sensitive, minimally invasive, and fast determination of drug concentrations in plasma constitutes a significant analytical problem in medicine.

The aforementioned requirements can be met by electrochemical sensors. Several studies were performed with the use of electrodes modified by carbon-based materials and azaporphyrins. Two examples concerned the detection of a common painkiller—acetaminophen. In one study, glassy carbon electrodes were modified by both gold nanoparticles and MWCNTs with unsubstituted cobalt(II) phthalocyanine embedded on their surface and revealed submicromolar limit of detection of acetaminophen [147]. In the second one, the surface of MWCNTs was covered by cobalt(II) phthalocyanine with flavone or benzoxazole groups, respectively, and the obtained nanomaterial was used to modify GC electrode. The LODs obtained in square wave voltammetry studies were of 1 and 15 µM, respectively (in wide linear range of 1–1000 and 15–1500 µM, respectively) [148].

A few other APIs, representing different fields of pharmacotherapy, were subjected to electrochemical determination by the use of electrodes modified with carbon-based materials and various phthalocyanines. The selected data are presented in Table 1.

Despite the active pharmaceutical ingredients designated for humans, there was an example of electrochemical determination of one veterinary drug—carbadox, an antimicrobial agent used in swine dysentery and bacterial enteritis in young swine [156]. In this case, the surface of a GC electrode was modified by poly(pyrrole)-graphene oxide-binuclear phthalocyanine cobalt(II) sulphonate and then the surface of the electrode was covered by molecularly printed polymer including quinoxaline-2-carboxylic acid as a recognition element of sensor. The fabricated electrode revealed a nanomolar (2.1 nM) limit of detection.

## 9. Other Analytes’ Determination

There are several examples of electrochemical sensing of analytes which cannot be easily classified in previous sections of this review. An electrochemical biosensor based on tetrasulfonated copper(II) phthalocyanine and graphene deposited on interdigitated electrodes was used as an electronic tongue to recognize distinct soil samples (sandy and clayey) with the use of impedance spectroscopy technique [157]. Also, the electrochemical oxidation of 2-mercaptoethanol is worth attention due to the fact that oxidation of thiols is of industrial importance. Two studies concerned on this issue, using Co(II)Pc embedded on SWCNTs [158] and tetrasulfonated Cu(II) Pc on MWCNTs [159]. In the second case, the carbon nanotubes were negatively charged by the assembling of cationic poly(diallyldimethylammonium chloride).

## 10. Conclusions

From the beginning of the second decade of the 21st century, significant progress in the electrochemical determination of various substances can be observed. The synthesis of new azaporphyrin-based sensing materials gave access to more specific sensors with fast signal responses and low detection limits. However, the studies concerning new modifications of working electrodes involving carbon-based materials are still ongoing due to the fact that more sophisticated techniques for preparation and also additional materials are being implemented.

In light of the studies that have been performed in the last 10 years, some conclusions can be drawn regarding the utilization of different azaporphyrins and different carbon-based nanomaterials. These conclusions are: (i) non-enzymatic electrodes revealed lower LOD in glucose sensing than enzymatic ones; (ii) non-covalent deposition of unsubstituted azaporphyrins has so far been the most common method of electrode fabrication; (iii) among various metal complexes of azaporphyrins, the cobalt and iron ones were abundant; (iv) there were just a few examples of utilization of metallic porphyrazines instead of phthalocyanines; and (v) in the studies performed in the last 15 years, carbon nanotubes were more popular CBNs used in the determination of most analytes except for glucose sensing, probably due to their easier accessibility.

In this review, we summarized the utilization of azaporphyrins embedded on graphene or carbon nanotubes in electrochemical sensing of diverse analytes. The purpose of the review has been to show the promising potential of biosensors in the chemical and pharmaceutical industry, in environmental protection, and healthcare. In addition it is worth mentioning that the use of graphene and its reduced form is increasing when compared with well-known single- and multi-walled carbon nanotubes. This is as a result of its double surface area compared to CNTs, higher chemical reactivity due to a larger number of edge planes per unit mass, higher electron mobility, and conductivity. In addition, the production of graphene sheets can be less expensive and can also be obtained without metallic impurities, which sometimes introduce an anomalous sensing response. Furthermore, its ease of processing and low hazard properties make it more feasible for commercial applications [5].

## Figures and Tables

**Table 1:** Examples of active pharmaceutical ingredients (APIs) subjected to electrochemical determination with the use of Pcs/CBNs modified electrodes.

Analyzed Drug	Type of CBM	Azaporphyrin Derivative	Type of Electrode	Reference
L-dopa	Multi-walled carbon nanotubes (MWCNTs)	Unsubstituted Fe(II)Pc and Co(II) Pc	Carbon-nanotube paste electrode	[149]
Diethylstilbestrol—DES(synthetic estrogen)	MWCNTs functionalized with gold nanoparticles	Unsubstituted Co(II)Pc	GCE	[150]
N-acetylcysteine	Graphene nanoflakes	Unsubstituted Co(II)Pc	Carbon paste electrode	[151]
Erythromycin	GO	Tetraamiono-substituted nickel(II) phthalocyanine	Indium tin oxide coated electrode (photoelectrochemical sensor)	[152]
Rifampicin	GO	Tetraamiono-substituted nickel(II) phthalocyanine	Indium tin oxide coated electrode (photoelectrochemical sensor)	[153]
Pyridoxine (vitamin B6)	MWCNTs	Unsubstituted Co(II)Pc	Pyrolytic graphite electrode (PGE)	[154]
Isoniazid	Graphene-functionalized oxidized MWCNTs	Unsubstituted Fe(II)Pc	GCE	[155]

## Data Availability

Not applicable.
